# Identification of biomarkers and immune microenvironment associated with pterygium through bioinformatics and machine learning

**DOI:** 10.3389/fmolb.2024.1524517

**Published:** 2024-12-11

**Authors:** Li-Wei Zhang, Ji Yang, Hua-Wei Jiang, Xiu-Qiang Yang, Ya-Nan Chen, Wei-Dang Ying, Ying-Liang Deng, Min-hui Zhang, Hai Liu, Hong-Lei Zhang

**Affiliations:** ^1^ Department of Ophthalmology, The Affiliated Hospital of Yunnan University, Second People’s Hospital of Yunnan Province, The Eye Hospital of Yunnan Province, The Eye Disease Clinical Medical Research Center of Yunnan Province, The Eye Disease Clinical Medical Center of Yunnan Province, Kunming, China; ^2^ Department of Ophthalmology, Wuhan Children’s Hospital, Tongji Medical College, Huazhong University of Science and Technology, Wuhan, China; ^3^ Department of Ophthalmology, Diqing Tibetan Autonomous Prefecture People’s Hospital, Diqing, Yunnan, China; ^4^ Center for Scientific Research, Yunnan University of Chinese Medicine, Kunming, China

**Keywords:** pterygium, RNA sequencing, bioinformatics, WCGNA, machine learning, immuno-infiltration

## Abstract

**Background:**

Pterygium is a complex ocular surface disease characterized by the abnormal proliferation and growth of conjunctival and fibrovascular tissues at the corneal-scleral margin. Understanding the underlying molecular mechanisms of pterygium is crucial for developing effective diagnostic and therapeutic strategies.

**Methods:**

To elucidate the molecular mechanisms of pterygium, we conducted a differential gene expression analysis between pterygium and normal conjunctival tissues using high-throughput RNA sequencing. We identified differentially expressed genes (DEGs) with statistical significance (adjust *p* < 0.05, |logFC| > 1). Enrichment analyses were performed to assess the biological processes and signaling pathways associated with these DEGs. Additionally, we utilized weighted correlation network analysis (WGCNA) to select module genes and applied Random Forest (RF) and Support Vector Machine (SVM) algorithms to identify pivotal feature genes influencing pterygium progression. The diagnostic potential of these genes was validated using external datasets (GSE2513 and GSE51995). Immune cell infiltration analysis was conducted using CIBERSORT to compare immune cell populations between pterygium and normal conjunctival tissues. Quantitative PCR (qPCR) was used to confirm the expression levels of the identified feature genes. Furthermore, we identified key miRNAs and candidate drugs targeting these feature genes.

**Results:**

A total of 718 DEGs were identified in pterygium tissues compared to normal conjunctival tissues, with 254 genes showing upregulated expression and 464 genes exhibiting downregulated expression. Enrichment analyses revealed that these DEGs were significantly associated with inflammatory processes and key signaling pathways, notably leukocyte migration and IL-17 signaling. Using WGCNA, RF, and SVM, we identified KRT10 and NGEF as pivotal feature genes influencing pterygium progression. The diagnostic potential of these genes was validated using external datasets. Immune cell infiltration analysis demonstrated significant differences in immune cell populations between pterygium and normal conjunctival tissues, with an increased presence of M1 macrophages and resting dendritic cells in pterygium samples. qPCR analysis confirmed the elevated expression of KRT10 and NGEF in pterygium tissues.

**Conclusion:**

Our findings emphasize the importance of gene expression profiling in unraveling the pathogenesis of pterygium. The identification of pivotal feature gene KRT10 and NGEF provide valuable insights into the molecular mechanisms underlying pterygium progression.

## Introduction

Pterygium, a multiple ocular surface disease, arises from abnormal proliferation and growth of conjunctival and fibrovascular tissues at the corneal scleral margin, subsequently invading the cornea (Singh et al., 1988; Guimares et al., 2022). This condition significantly impacts visual health, altering appearance, destabilizing the tear film, and inducing discomfort. Furthermore, pterygium severely affects corneal astigmatism, leading to decreased vision, and in extreme cases, can cause eye movement disorders and blindness (Chu et al., 2020). The prevalence of pterygium varies globally, from 0.074% in Saudi Arabia to 53% in Taiwan, China. Combined with global data (Alqahtani, 2013; Rezvan et al., 2018), the overall prevalence of pterygium is 12% (Rezvan et al., 2018). Numerous studies, both domestic and international, have established a strong correlation between ultraviolet radiation and the onset as well as progression of pterygium (Moran and Hollows, 1984; Modenese et al., 2023). There have also been studies on the relationship between surface air pollution and the publication of pterygium (Lu et al., 2023; Taylor, 2013). Epidemiological research further supports this finding, indicating a higher incidence among rural populations and individuals who engage in frequent outdoor activities, potentially due to increased exposure to ultraviolet radiation. The prevalence and postsurgical recurrence rate of pterygium are notably high, posing a significant threat to human visual health, as untreated cases may result in blindness (Prabhasawat et al., 1997; Chang et al., 2023). Surgical resection is the main treatment of pterygium. However, there are some serious complications in this treatment, such as postoperative recurrence and secondary infection (Hacıoğlu and Erdöl, 2017). Ocular demodicosis, environment pollution and ultraviolet radiation are risk factors for pterygium recurrence (Huang et al., 2013).

Current research on pterygium has largely focused on its clinical manifestations and surgical management. However, the molecular mechanisms underlying pterygium development remain poorly understood, creating a gap in knowledge that could inform future therapeutic strategies. In addition, there are also immune inflammation, cell proliferation and apoptosis disorders and lipid metabolism disorders and other related mechanisms involved in the development of pterygium (Shahraki et al., 2021; Chalkia et al., 2019; Kalogeropoulos et al., 2020; Rubeshkumar et al., 2020). Recent studies have highlighted the importance of inflammatory processes in pterygium pathology, revealing that the disease is associated with a heightened inflammatory response and altered immune cell infiltration within the conjunctival tissue (Labbé et al., 2010). This suggests that targeting inflammatory pathways may offer new avenues for intervention.

**FIGURE 1:**
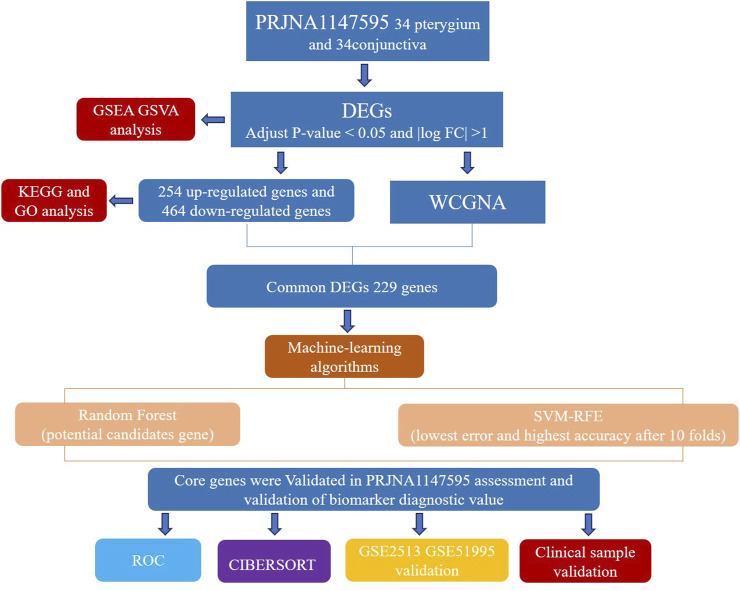
Flow chart of this study design.

Currently, there is a lack of consensus regarding the risk factors and clear molecular mechanisms underlying pterygium. Our study aims to contribute to a deeper understanding of pterygium by exploring these risk factors and potential molecular mechanisms. High-throughput RNA sequencing technologies have emerged as powerful tools for investigating gene expression profiles associated with various diseases, including pterygium. Studies employing RNA sequencing have demonstrated significant differences in gene expression between pterygium and normal conjunctival tissues, providing insights into the molecular pathways involved in pterygium development (Yoon et al., 2023). Furthermore, machine learning techniques such as random forests (RF) and support vector machines (SVM) have been applied to identify key gene signatures associated with pterygium, enhancing the predictive power of genomic data (Uddin et al., 2019). Figure 1 depicts our research protocol.

Therefore, an in-depth exploration of the molecular mechanisms underlying pterygium pathogenesis holds significant importance for disease prevention, treatment, and the identification of potential drug targets. Given the complexity and uncertainty surrounding the risk factors and molecular mechanisms of pterygium, we aim to screen for differentially expressed genes in pterygium and bulbar conjunctiva tissues through transcriptomics research. Additionally, we seek to identify module gene sets associated with the pterygium phenotype and analyze their biological functions. On a cellular level, we will assess differences in immune cell infiltration between pterygium and bulbar conjunctiva groups. This comprehensive approach not only advances our understanding of the molecular mechanisms of pterygium but also offers new insights into its transcription profile. Ultimately, this research strives to identify novel treatment methods and therapeutic targets, aiming to optimize disease prevention, treatment, and reduce the postoperative recurrence rate, thereby carrying substantial clinical significance.

## Materials and methods

### Patients and specimens

The research protocol was approved by the Ethics Review Committee of the Affiliated Hospital of Yunnan University, and all study participants gave written informed consent (approval number: 2022198). The study was carried out in accordance with the Declaration of Helsinki. All surgical procedures were performed under local anesthesia by the same surgeon. Thirty-four patients underwent elective pterygium surgery, including 5 males and 29 females. The average age was 56.8 years and the average duration of illness was 4.19 years (Supplementary Figure S1). During the surgical procedure, the affected pterygium tissue was removed alongside the surrounding excess loose conjunctiva. The integrity of the total RNA extracted from both pterygium and bulbar conjunctiva samples was evaluated using an Agilent 2,100 bioanalyzer. Subsequently, rRNA was eliminated from the total RNA to isolate the sample mRNA. This mRNA was then subjected to random fragmentation using divalent cations in NEB Fragmentation Buffer, followed by chain-specific fragmentation for mRNA construction. Initial library quantification was carried out with a Qubit2.0 Fluorometer, and the library was subsequently diluted to a concentration of 1.5 ng/ul. The size of the library inserts was determined using an Agilent 2,100 bioanalyzer, and QRT-PCR was utilized to accurately quantify the library’s effective concentration, which needed to exceed 2 nM. After assessing the quality of the genomic DNA, it was fragmented through mechanical interruption (ultrasound). The fragmented DNA then underwent purification, end-repair, 3′end adenylation, ligation to a sequencing adapter, and size-selection using agarose gel electrophoresis. The resulting polymerase chain reaction (PCR) product was amplified to generate the sequencing library. Sequencing was conducted on the Illumina NovaSeq 6,000 platform with a read length of 150 bp. Through quality control, trimming, deduplication, and alignment of the original fastq data by a high-throughput sequencing service provider, we obtained the gene expression matrix of transcriptome sequencing. The data presented in this publication has been archived in NCBI’s Sequence Read Archive (SRA) database (accession number: PRJNA1147595, URL: https://dataview.ncbi.nlm.nih.gov/object/PRJNA1147595?reviewer=3k3nnr66jke53qo77la1sbt91b). Supplementary datasets can be found in NCBI’s Gene Expression Omnibus (GEO) (accession numbers: GSE2513, URL: https://www.ncbi.nlm.nih.gov/geo/). GSE2513 includes four conjunctival samples and eight matched pairs of pterygium and control conjunctival samples (Wong et al., 2006). GSE51995 includes four conjunctival samples and four pairs of pterygium and control conjunctival samples (Hou et al., 2014).

### Detection of differentially expressed genes (DEGs) in conjunctiva versus pterygium samples

To detect DEGs, we employed the “DESeq2” R package. The DESeq2 package was used for difference analysis of the original Counts matrix, and the analysis was carried out according to the standard process. The Variance Stabilizing Transformations (VST) method provided by package DESeq2 was used to Normalize the original Counts matrix. A Wilcoxon rank sum test was conducted to assess gene expression differences between the conjunctiva and pterygium samples. Genes were considered significantly differentially expressed if they met the criteria of adjusted *p* < 0.05 and |logFC| >1. For data visualization, we utilized the “pheatmap”, “ggpubr” and “ggplot2” R packages.

### Functional annotation and enrichment analysis of DEGs

To analyze the DEGs, we employed the R tool known as “clusterProfiler.” (Yu et al., 2012) Using the “clusterProfiler” R package, we carried out both Gene Ontology (GO) (Ashburner et al., 2000) and Kyoto Encyclopedia of Genes and Genomes (KEGG) analyses (Ogata et al., 1999). Gene set enrichment analysis (GSEA) was conducted on all genes (previously ranked based on their log2FC between analyzed groups) using the cluster profiler package. Enrichment was considered significant if the nominal false discovery rate (FDR) was <0.25 and the P-value was <0.05, referencing the ‘c2. cp.all.v2022.1. Hs.symbols.gmt’ gene set. Additionally, we conducted Gene Set Variation Analysis (GSVA) by utilizing the “GSEABase” and “GSVA” R packages (Hänzelmann et al., 2013) and referencing the ‘h.all.v2023.2. Hs.symbols.gmt’ gene set. These packages offer utilities for evaluating the enrichment or variability of gene sets within gene expression data, thereby enabling a thorough examination of pathway or gene set activities across different samples. P-values were calculated with the Benjamini–Hochberg method, and the terms with P-values <0.05 were considered to be significant.

### Weighted gene co-expression network analysis

For the construction of expression networks within the PRJNA1147595 dataset, we utilized the weighted gene co-expression network analysis (WGCNA) approach. This method was steered by adhering to the scale-free topology criterion, ensuring that the resultant network displayed a scale-free structure characterized by a power-law distribution of node connections. To ascertain the appropriate soft threshold power and establish adjacencies, we employed the “pickSoftThreshold” function from the WGCNA package. Once determined, the adjacency matrix was transformed into a topological overlap matrix (TOM). Hierarchical clustering analysis was then conducted based on the dissimilarity derived from the TOM.

To delineate co-expressed gene modules, we adopted the dynamic tree cutting technique, setting a minimum module size of 50. This approach allowed us to categorize genes into distinct modules according to their expression patterns. Subsequently, we evaluated the relationship between these gene modules and the trait of interest, pterygium, by considering both gene significance (GS) and module membership (MM) values. GS reflects the correlation between gene expression and pterygium, whereas MM measures the extent of co-expression within a given module. By integrating GS and MM values, we pinpointed the key modules linked to pterygium, suggesting their potential functional importance in the context of the disease.

### Identification of hub genes based on machine learning methods

The gene sets of the significant difference modules analyzed by WCGNA were intersected with the expression difference genes obtained by DESeq2 analysis. To identify the key hub genes, two distinct machine learning algorithms were employed: support vector machine (SVM) and random forest (RF). The SVM algorithm was executed using the “e1071”package, offering a comprehensive toolkit for SVM model training and classification. The RF algorithm was applied using the “randomForest” package, where the error rate was computed across 1 to 500 trees. The optimal tree count was determined by selecting the value that minimized the error rate while ensuring stability. Subsequently, the RF classifier was leveraged to compute feature importance scores. This selection utilized the Gini coefficient method to pinpoint the most pertinent genes. The intersection of the top five feature genes from both the SVM and RF models was used to pinpoint crucial hub genes involved in the disease process.

Utilizing the R programming language, receiver operating characteristic (ROC) curves were generated for the hub genes. To evaluate the distinctive capabilities between pterygium tissues and conjunctiva tissues, the area under the curve (AUC) was calculated for the respective ROC curves of these hub genes.

### Analysis of immune cells infiltration

According to the CIBERSORT deconvolution algorithm (Newman et al., 2015), we analyzed the infiltration of 22 kinds of immune cells in pterygium and conjunctival tissues, and compared whether there was a significant difference between the two groups. To assess the degrees of immune cell infiltration in both conjunctiva and pterygium samples, we utilized the CIBERSORT technique. This method enabled us to measure gene expression levels quantitatively and ascertain the infiltration levels among patients. The immune cell populations encompassed both immune-stimulating and immune-inhibiting cells. To examine the relationships between these immune cells, we conducted a correlation analysis using the Spearman coefficient and depicted the results via correlation heatmaps. At the same time, we also analyzed the correlation between hub gene and immune cells. We considered findings statistically significant if they met the threshold of *p* < 0.05.

### Validation of the mRNA expression of hub genes by quantitative real-time PCR

Patient Sample Preparation. There were 4 primary pterygium specimens obtained in Affiliated Hospital of Yunnan University during pterygium surgery. The control conjunctival tissues were derived from healthy conjunctival tissues on the temporal side of the surgical eye of the same patient, with a size of 1.5 mm × 1.5 mm.

Tissue RNA Extraction and RT-qPCR. All specimens were collected during the operation and immediately placed in RNA protective agent, stored at −20°C for usage. RNA was extracted from 80 mg specimen tissue using TRIZOL reagent. The relative expression of hub genes was measured using a reverse kit (HiScript® III RT SuperMix for qPCR (+gDNA wiper)) and ChamQ™ Universal SYBR® qPCR Master Mix (Vazyme company, Nanjing, China) on CFX96 fluorescence qPCR instrument. And the PCR reaction was carried as follows: predenaturation at 95°C for 30 s, denaturation at 95°C for 3–10 s, and annealing extension at 60°C for 10–30 s, for 40 cycles. Using GAPDH as internal references, the relative expression of genes was calculated by the 2-△Ct method.

### Identification of key miRNAs and potential drug candidates

The identification of key miRNAs targeting the two characteristic genes, as well as the screening of potential drug candidates, was conducted through a rigorous and comprehensive process. We utilized the Targetscan database (version 7.2, accessed via the Enrichr platform at http://amp.pharm.mssm.edu/Enrichr/) to forecast miRNAs that could potentially target the selected genes (Agarwal et al., 2015). This involved inputting the gene symbols into the Targetscan interface and retrieving a list of predicted miRNAs based on the presence of conserved target sites within the 3′UTRs of the genes. The predicted miRNAs were then filtered based on their conservation across species, expression levels in relevant tissues, and previous reports of their involvement in related biological processes. Subsequently, to identify potential drug candidates that could modulate the expression or activity of these genes, we accessed the DSigDB database through the Enrichr platform. This database integrates drug signatures from various sources, allowing us to search for drugs that have been reported to affect the expression or activity of the selected genes.

### Statistical analysis

The statistical analyses were performed using R software (version 4.2.1). All *p* values were two-sided, and significance was indicated by *p* < 0.05.

## Results

### Identification of the DEGs

**FIGURE 2:**
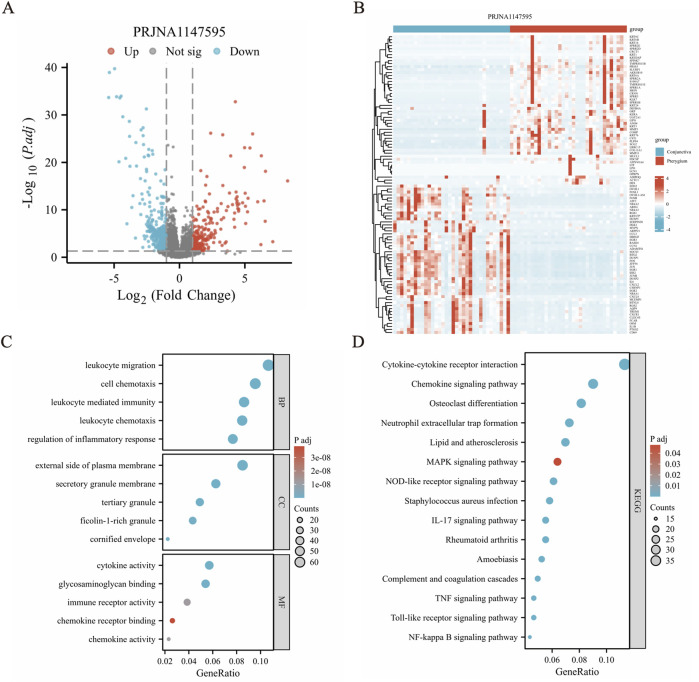
Differential gene expression analysis revealed significant variations between conjunctiva and pterygium. **(A)** Volcano diagram shows that there are 254 upregulated genes and 464 downregulated genes in the pterygium group compared with the conjunctival group, a total of 718 differentially expressed genes. **(B)** Cluster heat map shows the expression of the top 50 upregulated and downregulated genes. **(C)** GO functional enrichment analysis results. **(D)** Results of KEGG pathway enrichment analysis.

Based on the RNA-Seq data, a total of 718 DEGs were obtained, and these genes reached the threshold adjust *p*-value of <0.05 (Supplementary Table S1). Among these DEGs, 254 transcripts were upregulated in the disease group against the health one, while 464 transcripts were found to decrease in pterygium tissues (Figure 2A). In addition, the relative expression levels are shown in a heatmap plot and the top 50 upregulated and downregulated genes were shown using a volcano plot (Figure 2B).

### Gene function analysis for the DEGs

GO and KEGG pathway enrichment analyses were conducted to assess the function of these 718 DEGs. The clusterProfiler package was used for GO and KEGG pathway enrichment analysis to find potential biological pathways. The top 5 BP (biological process), CC (cellular component) and MF (molecular function) terms with the lowest *p*-values in each category were selected and visualized using bubble plots (Figure 2C). The DEGs were primarily enriched in BP terms such as “leukocyte migration (GO:0050900)”, “leukocyte chemotaxis (GO:0030595)”, “cell chemotaxis (GO:0060326)”, “leukocyte mediated immunity (GO:0002443)” and “regulation of inflammatory response (GO:0050727)”. Genes were also enriched in CC terms like “external side of plasma membrane (GO:0009897)”, “tertiary granule (GO:0070820)”, “secretory granule membrane (GO:0030667)”, “ficolin-1-rich granule (GO:0101002)” and “cornified envelope (GO:0001533)”. Additionally, the DEGs were enriched in MF terms including “cytokine activity (GO:0005125)”, “glycosaminoglycan binding (GO:0005539)”, “immune receptor activity (GO:0140375)”, “chemokine activity (GO:0008009)” and “chemokine receptor binding (GO:0042379)”. In the KEGG pathway enrichment results, many pathways related to inflammation and signal transduction were enriched, such as IL-17 signaling pathway (hsa04657), chemokine signaling pathway (hsa04062), TNF signaling pathway (hsa04668), MAPK signaling pathway (hsa04010) and cytokine-cytokine receptor interaction (hsa04060) (Figure 2D) and so on (Supplementary Table S2).

### GSEA and GSVA analysis

**FIGURE 3:**
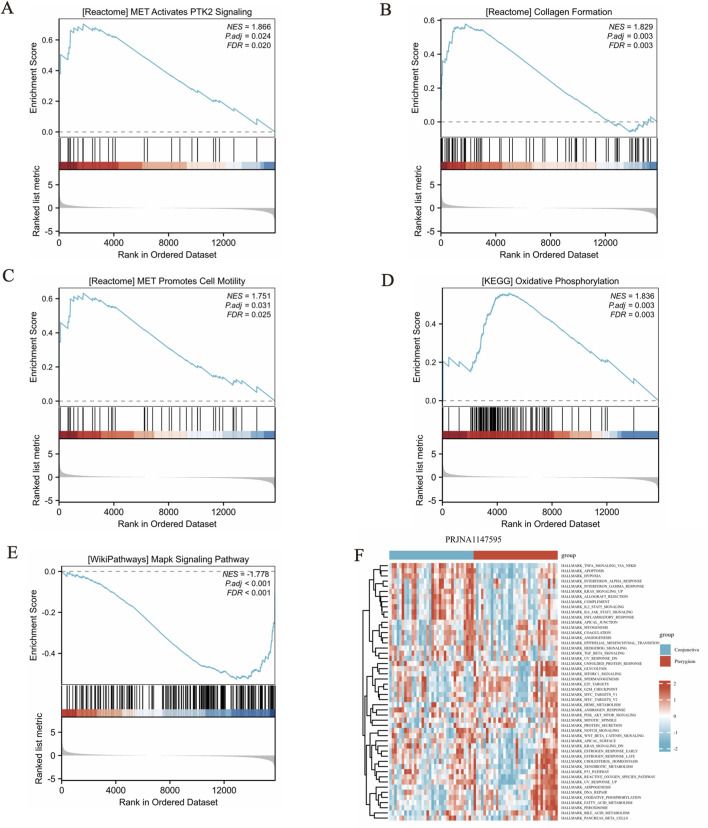
Gene set enrichment analysis (GSEA) and gene set variation analysis (GSVA) results. **(A-D)** The significant enrichment in upregulated pathways. The members of the line marker pathway gene set appear in the gene ranking list, and the bottom is the rank value distribution of all genes. **(E)** The significant enrichment in downregulated pathway. The results obtained from the GO database and the KEGG database for the pre-defined gene set. **(F)** The differential pathways in the dataset encompassed, showing in cluster heat map.

Our reference gene set was ‘c2. cp.all.v2022.1. Hs.symbols.gmt’. The datasets underwent GSEA enrichment analysis to identify significant enrichment based on the criteria of FDR <0.25 and *p* < 0.05. In gene set enrichment analysis (GSEA), the significant enrichment in upregulated pathways including REACTOME_MET_ACTIVATES_PTK2_SIGNALING, REACTOME_COLLAGEN_FORMATION, REACTOME_MET_PROMOTES_CELL_MOTILITY, KEGG_OXIDATIVE_PHOSPHORYLATION and so on (Figures 3A–D). The significant enrichment in downregulated pathways including WP_MAPK_SIGNALING_PATHWAY (Figure 3E) and so on (Supplementary Table S3). GSVA enrichment analysis was conducted on the PRJNA1147595 dataset, revealing distinct pathways (Supplementary Table S4). By utilizing the gene set variation analysis (GSVA) package and referencing the “h.all.v2023.2. Hs.symbols.gmt” gene set (Hänzelmann et al., 2013), the gene expression matrix data were subjected to GSVA. The GSVA variance analysis was performed using the limma package of R software. Differential pathways were filtered based on an *p*-value <0.05 and |log2FC| > 0.25 (Supplementary Table S4). The differential pathways in the PRJNA1147595 dataset encompassed HALLMARK_TNFA_SIGNALING_VIA_NFKB, HALLMARK_INFLAMMATORY_RESPONSE, HALLMARK_OXIDATIVE_PHOSPHORYLATION, HALLMARK_IL6_JAK_STAT3_SIGNALING and HALLMARK_ALLOGRAFT_REJECTION (Figure 3F).

### WGCNA analysis

**FIGURE 4:**
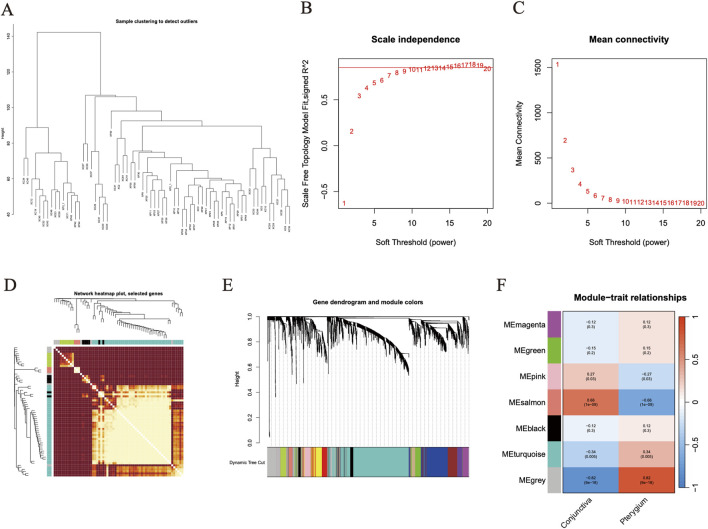
Weighted gene co-expression network analysis screened target module genes. **(A)** The figure is the gene clustering tree. **(B–C)** The relevant parameters of WGCNA network construction, at a power value of 11, an independence level of 0.9 was attained. **(D)** Clustering heat map of all module genes in WGCNA analysis. **(E)** Heat map of different phenotypes and module correlation analysis. Based on WGCNA analysis, the correlation between 7 gene modules and two phenotypes was obtained. **(F)** The expression of salmon module gene and turquoise module gene in the two groups was shown by cluster heat map. Red represents high expression of gene and blue represents low expression of gene.

Initially, expression data from 34 pterygium samples and 34 conjunctiva samples were utilized to establish co-expression modules through the WGCNA algorithm. We focused on the top 25% of genes exhibiting the greatest variability for subsequent investigation. Utilizing the “flashClust” package, we conducted cluster analysis on these samples, with the findings illustrated in Figure 4A. At a power value of 11, an independence level of 0.9 was attained, accompanied by an elevated mean connectivity (Figures 4B, C). By employing the dynamic cutting technique, we discerned seven unique gene co-expression modules within pterygium, and a heatmap of the TOM was also generated (Figures 4D, E). The genes within these seven modules were concurrently employed to explore the relationship between module eigengenes and clinical characteristics (Figure 4F). Notably, 3,956 genes were divided into 7 modules. Among them, salmon module (cor = −0.66, P = 1e-9) and turquoise module (cor = 0.34, *p* = 0.005) were significantly correlated with pterygium phenotype (Figure 4F).

### Identification of hub genes in pterygium using machine learning algorithms

**FIGURE 5:**
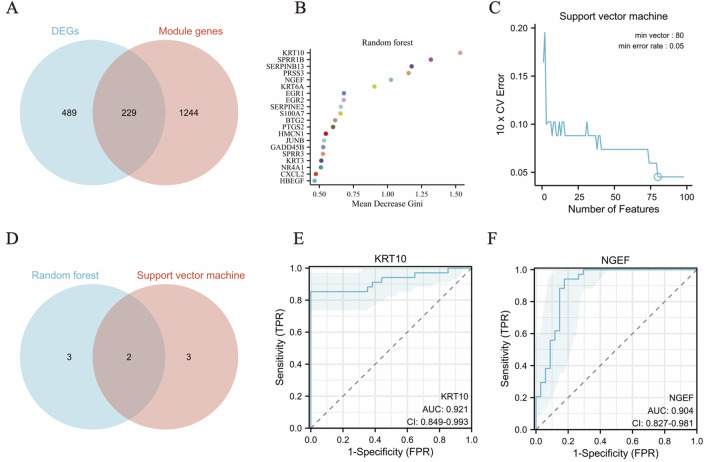
Machine learning algorithms identify biomarkers in pterygium. **(A)** The common genes in DEGs and the salmon and turquoise modules genes. **(B)** The top 20 feature genes selected by RF algorithm. **(C)** Error rate of SVM algorithm 10X cross validation. **(D)** The top five feature genes from both the SVM and RF models **(E–F)**. The ROC curve was evaluated the diagnostic efficiency of KRT10 and NGEF.

A Venn diagram was constructed to visually represent the relationships among the differentially expressed genes (DEGs) and the salmon and turquoise modules identified through Weighted Gene Co-expression Network Analysis (Figure5A). Subsequently, two distinct machine learning algorithms—SVM and RF—were leveraged to meticulously screen and pinpoint key signature genes within the pterygium dataset. For the RF classifier, an optimal number of 500 trees was selected, a decision grounded in an evaluation of error rates and classifier stability across varying tree counts. By analyzing the Mean Decrease Gini results, we identified top five pivotal hub genes: KRT10, SPRR1B, SERPINB13, PRSS3and NGEF (illustrated in Figure 5B). The SVM algorithm was also employed to rigorously select hub genes associated with pterygium, revealing five significant genes: KRT10, KRT6B, BTG2, ASPG and NGEF (Figure 5C).

To identify crucial hub genes involved in pterygium, we examined the intersection of the top five feature genes from both the SVM and RF models (Figure 5D). The model’s performance was evaluated using the area under the ROC curve (AUC) (Figures 5E, F). Impressively, the dataset yielded an AUC of 0.921 (KRT10) and 0.904 (NGEF), indicating a high level of accuracy in classifying gene expression data. Notably, KRT10 and NGEF were consistently identified as hub genes associated with pterygium across all two machine learning methods and WGCNA.

We downloaded the original data of GSE2513 and GSE51995 from GEO website, and conducted probe annotation and data standardization on the original expression data to obtain the gene expression matrix. The datasets GSE2513 and GSE51995 were merged utilizing the R packages dplyr and purrr, and the shared genes were identified based on the intersection of genes derived from sequencing analysis. Subsequently, the cleaned data were standardized using the stats, preprocessCore, and limma R packages. The resultant standardized data were then subjected to further analysis (Smyth and Gentleman, 2013). KRT10 achieved an AUC of 0.906 in GSE2513 and GSE51995 combined data and NGEF achieved an AUC of 0.625 (Supplementary Table S2).

### Immuno-infiltration landscape between conjunctiva and pterygium

**FIGURE 6:**
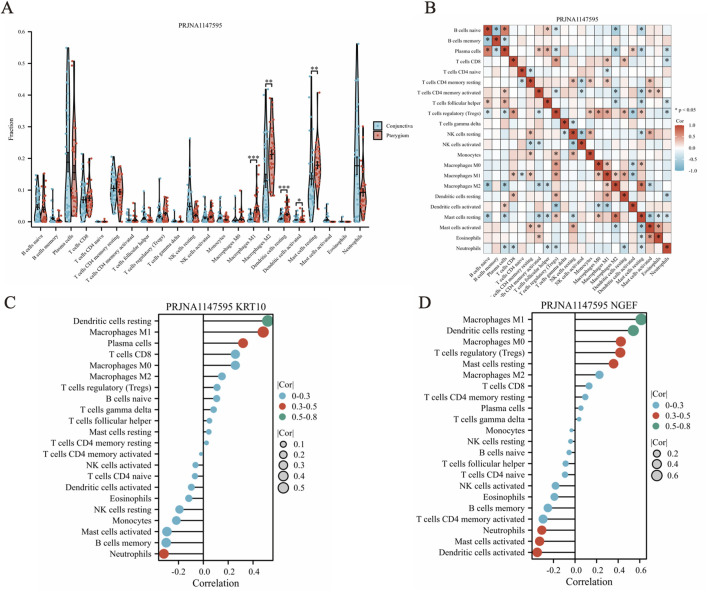
CIBERSORT algorithm was used to analyze the infiltration of 22 kinds of immune cells in pterygium and conjunctival tissues. **(A)** The abundance of 22 immune cell types in pterygium and conjunctival samples. **(B)** Correlation analysis between 22 kinds of immune cells in pterygium samples. **(C–D)** The correlation between KRT10 and NGEF genes and immune cells in pterygium group.

The CIBERSORT deconvolution algorithm was employed to quantify the infiltration of 22 distinct immune cell types within pterygium and bulbar conjunctiva tissues (Figure 6A). Our analysis revealed significant differences in the levels of five immune cells between these two tissue types. Specifically, M1 macrophages, resting dendritic cells, and activated dendritic cells were notably elevated in the pterygium group, whereas resting mast cells and M2 macrophages were significantly increased in the bulbar conjunctiva group (Figure 6A). Furthermore, a general correlation was observed among the 22 immune cell types within pterygium samples (Figure 6B). Correlation analysis between these immune cells and KRT10 (a hub gene) in pterygium tissues indicated that dendritic resting cells (cor = 0.58, *p* = 1.63e-07) and M1 macrophages (cor = 0.43, *p* = 3.21e-05) were positively correlated with KRT10 expression, while Neutrophils also exhibited a significant negative correlation with KRT10 (cor = −0.38, *p* = 0.0016) (Figure 6C). Correlation analysis between these immune cells and NGEF in pterygium tissues indicated that Macrophages M1 (cor = 0.61, P = 2.57e-08), Dendritic cells resting (cor = 0.54, P = 1.95e-06), Macrophages M0 (cor = 0.42, P = 0.0003), T cells regulatory (cor = 0.42, P = 0.0004) and Mast cells resting (cor = 0.36, P = 0.0029) were positively correlated with NGEF expression, while Dendritic cells activated (cor = −0.35, *p* = 0.0034) and Mast cells activated (cor = −0.33, *p* = 0.0063)exhibited a significant negative correlation with NGEF (Figure 6D).

To validate our findings, we employed the combined dataset of GSE2513 and GSE51995 from the GEO database as a verification cohort. Following CIBERSORT analysis on this integrated dataset, we observed that there were no statistically significant differences in the proportions of M1 macrophages, resting dendritic cells, activated dendritic cells, resting mast cells, and M2 macrophages between the pterygium and conjunctiva groups. Additionally, a comprehensive correlation analysis revealed notable associations among the 22 immune cell types within the pterygium samples (Supplementary Table S3).

### Validation of the mRNA expression of hub genes by quantitative real-time PCR

**FIGURE 7:**
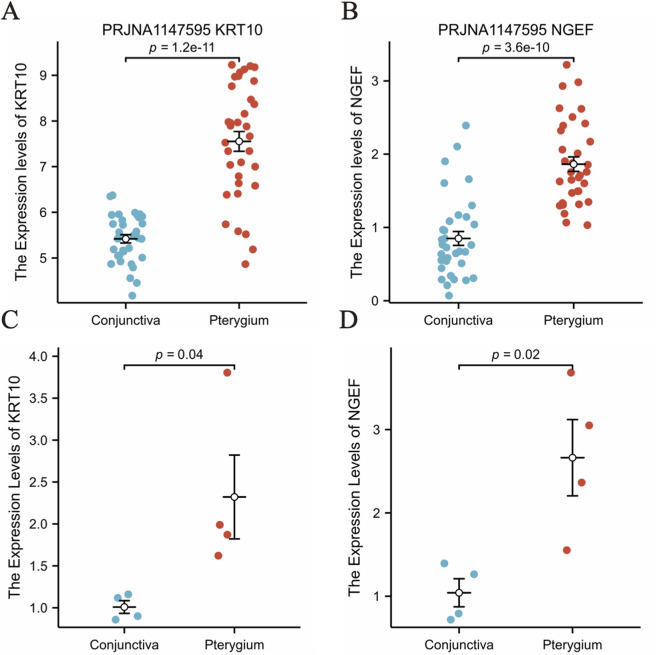
Verification of KRT10 and NGEF genes expression. **(A–B)** The expression of KRT10 and NGEF in the pterygium group and the conjunctival group was compared in our RNA-seq data. **(C–D)** The expression of KRT10 and NGEF in 4 pairs of pterygium and conjunctival tissues was analyzed.

In our analysis of RNA-Seq data derived from 68 samples, we observed a statistically significant elevation in the expression levels of KRT10 and NGEF genes in the pterygium group compared to the bulbar conjunctiva group, with a P-value <0.05 (as depicted in Figures 7A, B). To validate these findings, we conducted a qPCR experiment, which confirmed a significantly higher expression of KRT10 and NGEF in the pterygium group relative to the bulbar conjunctiva group, with a P-value of 0.04 and 0.02 as illustrated in Figures 7C, D and primer sequences are shown in Supplementary Table S5.

### Identification of key miRNAs and potential drug candidates

**FIGURE 8:**
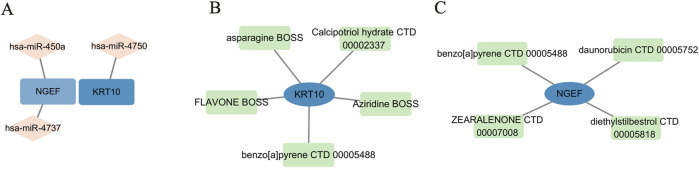
MiRNA-mRNA and drug-gene network construction. **(A)** 3 Pivotal miRNAs targeting 2 feature genes. **(B–C)** Candidate drug molecules targeting 2 feature genes.

To pinpoint crucial miRNAs and potential drug candidates that target the two identified feature genes, we conducted a comprehensive analysis using data sourced from the Targetscan and DSigDB databases. This rigorous screening process, employing a statistical significance threshold of *p* < 0.05, led to the identification of three pivotal miRNAs (depicted in Figure 8A). Our findings revealed intricate interaction networks: KRT10 was found to interact with one distinct miRNA (hsa-miR-4750), while NGEF was associated with hsa-miR-450a and hsa-miR-4737.

Furthermore, we undertook an extensive screening to identify the drug molecules, guided by P-value < 0.05 as per the DSigDB database (illustrated in Figures 8B, C). We conducted a rigorous screening process and identified KRT10 and NGEF as crucial genes implicated in the pathogenesis of pterygium. Subsequently, we presented only the top four drugs that significantly influenced KRT10 expression, along with benzo [a] pyrene CTD 00005488, which demonstrated the ability to jointly regulate both KRT10 and NGEF genes. In the case of the NGEF gene, our analysis revealed only four drugs with statistical significance (*p* < 0.05), including benzo [a] pyrene CTD 00005488, and we have accordingly reported these four drugs. Among these, benzo [a]pyrene (CTD 00005488) emerged as a prominent candidate, demonstrating interactions with all two feature genes and achieving a notable combined score of 94002.9. The remaining drug candidates exhibited interactions with KRT10 and NGEF, offering valuable insights for the advancement of pterygium treatment research and development.

## Discussion

Pterygium, a prevalent ophthalmologic condition, is distinguished by abnormal proliferation of subconjunctival fibrous tissue accompanied by angiogenesis, ultimately invading the central cornea. This condition derives its name from its resemblance to insect wings (Lan et al., 2018). The clinical management of pterygium primarily involves surgical excision; however, the high recurrence rates following surgery remain a significant challenge, necessitating further exploration of its underlying pathophysiological mechanisms (Rim et al., 2017).

Our study aims to delve into the molecular underpinnings of pterygium by investigating the differential gene expression between pterygium-affected and normal conjunctival tissues. Utilizing high-throughput RNA sequencing coupled with bioinformatics analyses, we identified 718 differentially expressed genes (DEGs) that may elucidate the biological processes involved in pterygium pathogenesis. Of particular note is the stark contrast between the upregulated (254) and downregulated (464) genes, suggesting that the pathogenesis of pterygium involves extensive dysregulation of gene expression. The biological functions of these DEGs warrant further exploration, especially concerning their roles in cellular proliferation, inflammation, and tissue remodeling. For instance, genes associated with inflammatory responses, such as those implicated in the IL-17 signaling pathway, could elucidate the chronic inflammatory nature of pterygium development. Additionally, the distinct gene expression profiles identified through heatmaps and volcano plots could serve as a foundation for developing targeted therapies. Importantly, the use of Weighted Correlation Network Analysis (WGCNA) in conjunction with machine learning algorithms like Random Forest (RF) and Support Vector Machine (SVM) to pinpoint key feature genes such as KRT10 and NGEF exemplifies a robust methodological framework that enhances the reliability of the findings, thereby paving the way for subsequent biological validation and clinical application (Lindsay and Sullivan, 2001; Ono et al., 2021).

The pathway enrichment analysis, encompassing Gene Ontology (GO) and Kyoto Encyclopedia of Genes and Genomes (KEGG), underscores the significant involvement of inflammation-related pathways in pterygium pathology. The enrichment in leukocyte migration and the IL-17 signaling pathway suggests that immune dysregulation plays a pivotal role in the disease’s etiology. This aligns with previous findings that highlight the involvement of inflammatory mediators in ocular surface diseases, indicating that targeting these pathways could represent a novel therapeutic strategy to mitigate pterygium progression. Future interventions could focus on modulating these inflammatory pathways to improve patient outcomes, particularly for those with recurrent disease. Furthermore, the integration of Gene Set Variation Analysis (GSVA) and Gene Set Enrichment Analysis (GSEA) results could facilitate a deeper understanding of the temporal dynamics of these pathways during disease progression (Zhang et al., 2024; Gupta et al., 2024). Pterygium is a chronic inflammatory proliferative lesion (Zhang et al., 2011). The CIBERSORT analysis revealing significant differences in immune cell infiltration, particularly the elevated levels of M1 macrophages and resting dendritic cells in pterygium tissues, emphasizes the immune landscape’s complexity (Zhao et al., 2023). The presence of pro-inflammatory M1 macrophages suggests a sustained inflammatory response, which could exacerbate tissue damage and drive disease progression. The interaction between various immune cell types and their roles in modulating the tumor microenvironment merits further investigation (Labbé et al., 2010; Celeva et al., 2011).

The validation of KRT10 and NGEF expression through quantitative PCR further solidifies the credibility of the RNA-Seq findings. This experimental confirmation not only reinforces the role of these DEGs in pterygium but also suggests their potential utility as biomarkers for disease diagnosis and prognosis (Adiguzel et al., 2007; March et al., 2019). The identification of potential drug molecules interacting with KRT10 and NGEF, such as benzo [a]pyrene, opens new avenues for therapeutic development (Zhang et al., 2022). These findings underscore the importance of drug repurposing strategies that leverage existing pharmacological agents to target specific pathways implicated in pterygium. The ability to identify compounds that modulate the expression or activity of key genes could lead to more effective treatment modalities. Future research should focus on the pharmacodynamics and pharmacokinetics of these identified compounds in the context of pterygium, as well as their safety profiles in clinical populations.

This study acknowledges several limitations that warrant consideration. Firstly, the sample size utilized in our analysis was relatively modest, which may restrict the generalizability of our findings. A larger cohort would enhance the robustness of our conclusions and facilitate a more comprehensive understanding of the genetic landscape associated with pterygium. Secondly, the absence of long-term clinical follow-up limits our ability to assess the prognostic implications of the identified biomarkers. Additionally, batch effects inherent in high-throughput sequencing could influence the consistency of gene expression profiles, potentially confounding the interpretation of results. Lastly, while we have established a correlation between differential gene expression and disease pathology, the lack of experimental validation for all findings presents a challenge in confirming causative relationships. Future studies should aim to address these limitations through larger, longitudinal investigations and rigorous experimental validations to bolster the reliability of our insights.

## Conclusion

In conclusion, this study offers an exhaustive examination of the molecular mechanisms underlying pterygium, emphasizing distinct gene expression profiles and their diagnostic and therapeutic implications. The discovery of pivotal genes, namely, KRT10 and NGEF, coupled with insights into immune cell infiltration patterns and pertinent signaling pathways, establishes a foundation for future research endeavors aimed at deciphering the pathophysiology of pterygium. Moreover, the identification of potential pharmacological agents that interact with these key genes presents promising opportunities for the development of novel therapies. As we progress, it is crucial for subsequent studies to validate these findings in larger and more heterogeneous populations. This will ultimately facilitate the development of targeted interventions and personalized treatment strategies for patients suffering from pterygium.

## Data Availability

The datasets presented in this study can be found in online repositories. The names of the repository/repositories and accession number(s) can be found in the article/Supplementary Material.
