# Supplementary figures and images for: Identification of biomarkers and immune microenvironment associated with pterygium through bioinformatics and machine learning
Source: Front Mol Biosci. 2024 Dec 11;11:1524517. doi: 10.3389/fmolb.2024.1524517 (PMC11668640; doi:10.3389/fmolb.2024.1524517)

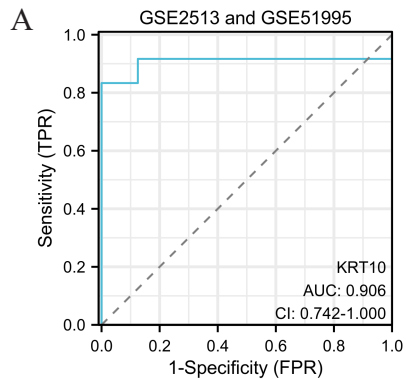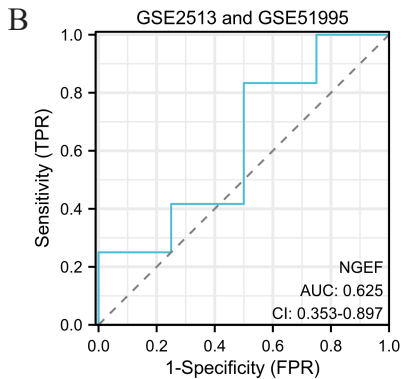

Supplement: Supplementary file 3 [file Image2.pdf]

A

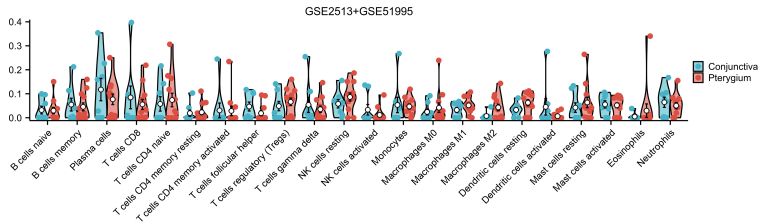

B

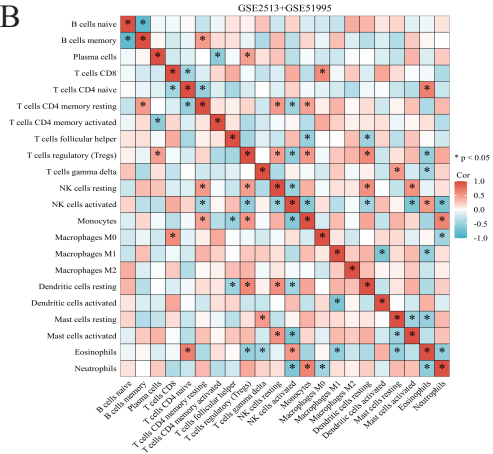

Supplement: Supplementary file 4 [file Image3.pdf]

A

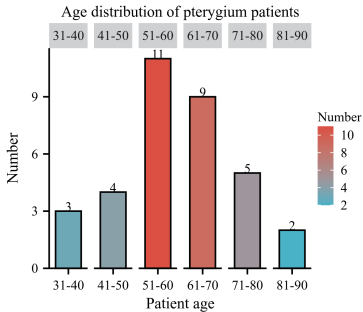

B

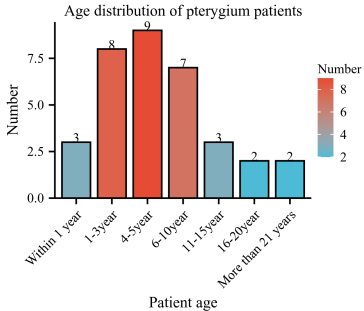

Supplement: Supplementary file 8 [file Image1.pdf]
